# Sesamin Protects against APAP-Induced Acute Liver Injury by Inhibiting Oxidative Stress and Inflammatory Response via Deactivation of HMGB1/TLR4/NF*κ*B Signal in Mice

**DOI:** 10.1155/2023/1116841

**Published:** 2023-08-24

**Authors:** Hui Du, Shiwen Tong, Ge Kuang, Xia Gong, Ningman Jiang, Xian Yang, Hao Liu, Nana Li, Yao Xie, Yang Xiang, Jiashi Guo, Zhenhan Li, Yinglin Yuan, Shengwang Wu, Jingyuan Wan

**Affiliations:** ^1^Department of Pharmacology, Chongqing Medical University, Chongqing, China; ^2^Department of Clinical Nutrition, The Second Affiliated Hospital of Chongqing Medical University, Chongqing 400016, China; ^3^Department of Anatomy, Chongqing Medical University, Chongqing, China; ^4^Chongqing Traditional Chinese Medicine Hospital, Chongqing, China; ^5^Clinical Immunology Translational Medicine Key Laboratory of Sichuan Province, Sichuan Provincial People's Hospital, University of Electronic Science and Technology of China, Chengdu, China; ^6^Department of Hematology, Xinqiao Hospital, Army Medical University, Chongqing, China

## Abstract

Acetaminophen (APAP) overdose would lead to liver toxicity and even acute liver failure in severe cases by triggering an inflammatory response and oxidative stress. Sesamin has been reported to possess anti-inflammatory and antioxidant actions in several animal disease models. In the present study, the effects and mechanisms of sesamin on APAP-induced acute liver injury (ALI) were explored. The results showed that pretreatment with sesamin significantly alleviated APAP-induced ALI, as indicated by decreased serum aminotransferase activities, hepatic pathological damages, and hepatic cellular apoptosis. But sesamin has no significant effects on the expression of cytochrome P450 2E1 (CYP2E1), APAP-cysteine adducts (APAP-CYS) production, and glutathione content in the liver of APAP-administered mice. Moreover, APAP-induced liver oxidative stress and inflammatory response also were remarkedly attenuated by sesamin, including reducing hepatic reactive oxygen species levels, promoting antioxidant generation, and inhibiting the expression of TNF-*α* and IL-1*β*, as well as decreasing inflammatory cell recruitment. Notably, sesamin inhibited serum high-mobility group box 1 (HMGB1) releases and blocked hepatic activation of Toll-like receptor 4 (TLR4)-interleukin 1 receptor-associated kinase 3-nuclear factor kappa B (NF-*κ*B) signaling pathway in APAP-administered mice. These findings indicated that sesamin could mitigate APAP-induced ALI through suppression of oxidative stress and inflammatory response, which might be mediated by the deactivation of HMGB1/TLR4/NF-*κ*B signaling in mice.

## 1. Introduction

Acetaminophen (APAP), also known as paracetamol, is one of the most popular over-the-counter pain relievers and antipyretics. However, it often involves unintentional overuse; excessive or long-term use would lead to liver toxicity and even induce acute liver failure (ALF) in severe cases [1–3]. Accumulating studies have shown that APAP hepatotoxicity is mainly related to the formation of electrophilic reactive metabolic intermediates *in vivo*, resulting in the induction of oxidative stress and mitochondrial function damage [4–6]. It has been reported that a small amount of APAP is metabolized by hepatic drug enzyme, mainly cytochrome P450 2E1 (CYP2E1), to reactive electrophilic oxidant N-Acetyl-P-Benzoquinone Imine (NAPQI) by when in a therapeutic dose, which is further detoxified by binding to glutathione (GSH) [7–11]. GSH spontaneously undergoes enzymatic reaction with APAP (only about 5%–10%) through GSH-S-transferase to form APAP-GSH glycine conjugate; most of APAP-GSH glycine conjugate is excreted in bile or urine [12]. Typically, detoxification of NAPQI involves binding GSH. However, after an overdose of APAP, the formation of NAPQI exceeds the detoxification ability of GSH, hepatotoxicity occurs when 70% of GSH is depleted in animal tests, and it will covalently bind with the protein sulfide group to form the APAP-cysteine protein adduct (APAP-CYS), eventually leads to mitochondrial dysfunction and oxidative stress to induce hepatocyte necrosis [13, 14]. Furthermore, the necrotic hepatocytes release a large number of damage-associated molecular patterns (DAMPs), including the protein high-mobility group box 1 (HMGB1) and mitochondria-derived DNA (mtDNA), which then activates innate immunity [15]. DAMPs bind to Toll-like receptors (TLRs) and activate the nuclear factor kappa B (NF-*κ*B) signaling pathway. Finally, excessive proinflammatory cytokines (IL-1*β*, TNF-*α*) and chemokines are produced and then cause an uncontrol inflammatory response. At present, there is no clinically specific drug for acute liver injury (ALI) induced by APAP.

**Figure 1:**
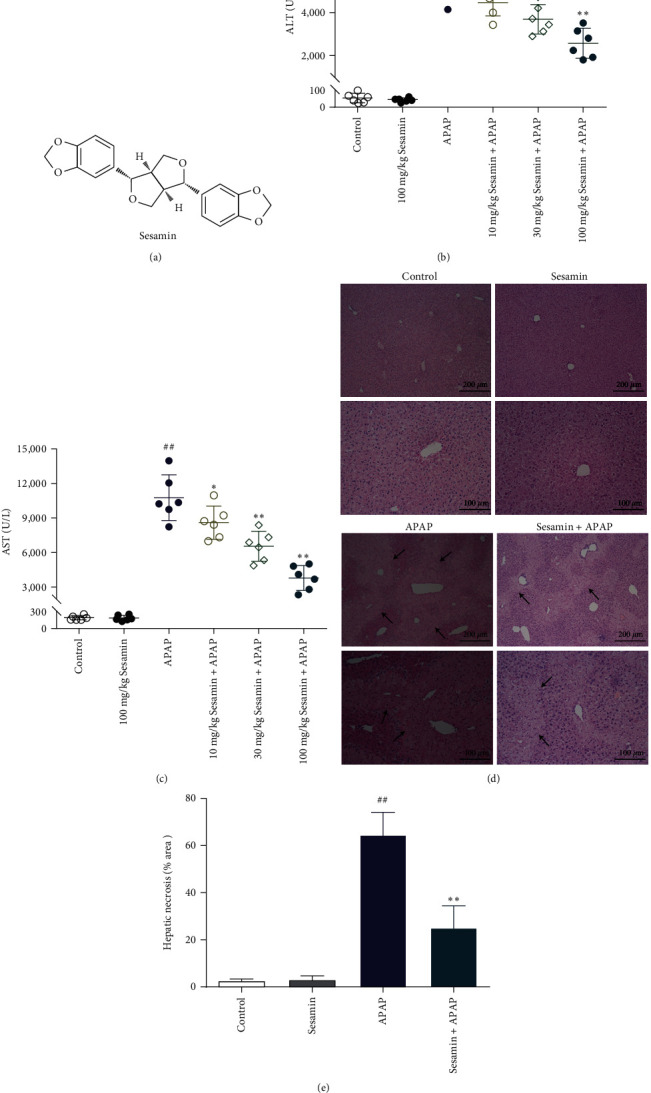
Sesamin attenuated APAP-induced acute liver injury in mice: (a) sesamin structural formula; (b) serum ALT activity; (c) serum AST activity; (d) hepatic H&E staining, black arrows indicate necrotic hepatocytes; (e) quantitative analysis of hepatic necrosis. Data were expressed as mean ± SD; sesamin group mice were oral gavage with 100 mg/kg sesamin, APAP group mice were intraperitoneally injected with 350 mg/kg APAP for 24 hr, sesamin + APAP group mice received oral gavages of sesamin 100 mg/kg at 16.5, 8.5, and 0.5 hr for three times before 350 mg/kg APAP administration (biological repeats *n* = 6), ^##^*P* < 0.01 compared with the control group,  ^*∗*^*P* < 0.05,  ^*∗∗*^*P* < 0.01, compared with APAP group.

Sesamin (Figure 1(a)), a sesame lignan derived from sesame seeds, has been shown to exhibit antihypertensive, antioxidant, cancer-preventative, and anti-inflammatory actions [16–18]. Previous studies have found that sesamin has protective effects on liver injury caused by different models, such as CCl_4_, LPS, and ischemia-reperfusion (I/R) liver injury [19–21]. Mechanically, sesamin could inhibit NF-kB activation, limit leukocyte infiltration, and reduce intercellular cell adhesion molecule-1 (ICAM-1) and TNF-*α* production *in vitro* and *in vivo* models [22, 23]. Our previous study found that sesamin could alleviate hepatic oxidative damage and apoptosis in mice exposed to CCl_4_ [20]. Wang et al. [24] found that sesamin also significantly inhibited hepatic I/R-induced IL-6 and IL-1*β* levels in serum. Based on the above, we hypothesize that sesamin may protect mice against ALI caused by APAP, and the proposed mechanism may be involved in the deactivation of innate immune TLR4 signaling, which decreases excessive oxidative stress and inflammatory response.

In this study, using an overdose APAP hepatotoxicity animal model, we explored the effect and underlying mechanism of sesamin on APAP-induced ALI. These findings may support the development of sesamin as a potent therapeutic agent for the management of liver damage induced by APAP overdose.

## 2. Materials and Methods

### 2.1. Reagents and Antibodies

Sesamin (purity > 98%) was supplied by Aladdin Industrial Corporation (Shanghai, China). APAP was provided by Sigma-Aldrich (St. Louis, MO, USA). The alanine aminotransferase (ALT), aspartate aminotransferase (AST), GSH, malonaldehyde (MDA), superoxidase dismutase (SOD), catalase (CAT) detection kits were purchased from Nanjing Jiancheng Bioengineering Company (Nanjing, China). Dihydroethidium (DHE) fluorescent probe, 3-nitrotyrosine antibody, TLR4 antibody, IRAK-1, and Phospho-IRAK-1 antibodies were purchased from Abcam (Cambridge, MA, USA). The antibodies for total and phosphorylated I*κ*B and p65 and GAPDH antibodies were purchased from Santa Cruz Biotechnology (Santa Cruz, CA, USA). The terminal deoxynucleotidyl transferase-mediated nucleotide nick-end labeling (TUNEL) kit was purchased from Roche Applied Science (Mannheim, Germany).

The caspase 3, 8, and 9 activity assay kits were from Beyotime Biotechnology (Shanghai, China). Trizol reagent was obtained from Invitrogen company (CA, USA). PrimeScript RT kit was purchased from Takara (Dalian, China). Radio Immunoprecipitation Assay (RIPA) lysis buffer was obtained from Beyotime company (Shanghai, China). The Halt Protease and Phosphatase Inhibitor Single-Use Cocktail and BCA protein detection kit was from ThermoFisher Scientific Inc. (Rochford, USA). CD11b-APC, F4/80-FITC, and Ly6G-PE antibodies were obtained from BioLegend Company (San Diego, CA, USA). The Fixable Viability Dye eFluor™ 450 was from ThermoFisher Scientific Inc. (Rochford, USA). The enzyme-linked immunosorbent assay (ELISA) kit for HMGB1 was purchased from CUSABIO Company (Houston, USA). The ELISA kits for TNF-*α*, IL-1*β*, and IL-10 were obtained from BD Biosciences (San Diego, USA).

### 2.2. Animals and Treatment

The male C57BL/6J mice (6–8 weeks old, 18–22 g) were provided from the Experimental Animal Center of Chongqing Medical University (Chongqing, China). The experimental animals were kept in a certain set of conventional laboratory conditions (between 20 and 25°C, 55% and 5% relative humidity, and a cycle of 12 hr of light and darkness). Before use, all mice underwent at least a one-week acclimatization period. The animal care and use committee of Chongqing Medical University's requirements were followed when conducting the studies using mice.

Mice were fasted for 12 hr but had free access to water before APAP hepatotoxicity. APAP was dissolved in warm normal saline, and mice were intraperitoneally injected with 350 mg/kg body weight APAP to induce ALI. Mice were received oral gavages of varying sesamin (10, 30, and 100 mg/kg, respectively) at 16.5, 8.5, and 0.5 hr for three times before APAP administration. Preliminary studies and other literature findings were used to determine the dose of sesamin used in this study [21, 25, 26]. The mice were slaughtered at 24 hr after APAP, and blood samples and liver tissues were collected to perform the next experiments.

### 2.3. Analysis of Liver Enzymes

Serum activities of ALT and AST were assessed utilizing standard enzymatic procedures according to the manufacturers' instructions as indicators for the evaluation of hepatocyte damage.

### 2.4. Analysis of Histopathology

The liver tissues that had been soaked in 4% paraformaldehyde for 48 hr were dehydrated and then embedded in paraffin. Tissue sections were stained with hematoxylin and eosin (H&E) and evaluated with an optical microscope. Three representative images per group were selected randomly for pathological quantitative analysis by ImageJ (https://imagej.net/software/imagej/#publication).

### 2.5. Detection of Apoptosis

Sections of tissue were dewaxed with xylene and hydrated with an ethanol gradient, the liquid around the sample was dried after protease K incubation; the TdT-mediated dUTP nick end labeling reaction mixture was dropwise added, then HRP-labeled secondary antibody before DAB solution was added after washing. Finally, the sections were dehydrated and sealed by neutral balsam mounting medium and then evaluated with an optical microscope for analysis. Three representative images per group were selected randomly for quantitative analysis by ImageJ. The activities of caspase-3, -8, and -9 in the liver were detected according to the manufacturer's instructions, respectively. Briefly, 100 *μ*L of lysate was added to 100 *μ*g of liver tissue and homogenized with a glass homogenizer on an ice bath. Then transfer the homogenate to a 1.5 mL centrifuge tube and lyse in an ice bath for 5 min. Centrifuge at 4°C 16,000 *g* for 10 min. Transfer the supernatant to a centrifuge tube precooled in an ice bath. Mix well after adding Ac-IETD-pNA (2 mM), taking care to avoid air bubbles during mixing. Incubate at 37°C for 120 min. Assay with a microplate reader at 405 nm immediately.

### 2.6. Measurement of APAP-Cysteine Adducts by HPLC-ECD

The liver homogenates were obtained by ultrasound and passed through the sodium acetate liquid dialysis for 30 hr (3,500 kDa interceptive distance). The homogenate sample was treated with trichloroacetic acid to precipitate the residual protein. Then, the sample was centrifuged at 4°C, 16,000 *g* for 10 min. The upper liquid is filtered by polyvinylidene difluoride. Microfuge filter tubes were immediately and slightly separated filter pipes before analysis by high-performance liquid chromatography with electrochemical detection (HPLC-ECD) method as previously reported [27].

### 2.7. Detection of Reactive Oxygen Species (ROS), GSH, MDA, SOD, and CAT

ROS levels in frozen sections of the liver were observed using DHE fluorescent probe. DHE itself is a blue probe with cell membrane permeability (Ex/Em:370/420 nm). Once DHE enters the cell, it will react with superoxide anion to generate 2-hydroxyethidium. 2-Hydroxyethidium produces red fluorescence (Ex/Em:535/610 nm) after inserting nucleic acid. Briefly, DHE was incubated in the dark at 4°C for 30 min. After rinsing with Tris-buffered saline, nuclear counterstained with 4,6-diamino-2-phenyl indole for 30 min at ambient temperature and in the dark. The liver sections were evaluated using a fluorescence microscope (Nikon, Japan). The contents of GSH, MDA, SOD, and CAT in the liver were measured using commercial kits according to the manufacturer's instructions, respectively.

### 2.8. Immunohistochemical Staining

For 3-NT detection, tissue slices were hydrated using an ethanol gradient after being dewaxed with xylene, and the liquid around the sample was dried after citric acid antigen repair. After bovine serum albumin (BSA) blocking, 3-NT antibody was added on the slice, then an HRP-labeled secondary antibody before the DAB solution was added. Eventually, the sections were dehydrated and sealed; an optical microscope was used to evaluate it.

### 2.9. Reverse Transcription-Quantitative Polymerase Chain Reaction (RT-qPCR)

Total RNA from the liver was extracted using the Trizol kit. Briefly, a homogenizer was used to combine 1 mL of Trizol reagent with 100 mg of liver tissue. The lysed liver sample was incubated for 5 min to allow the nucleoproteins to separate, then 200 *μ*L chloroform was added and mixed. The tube was tightly closed to continue incubating for 2 min. The sample was centrifuged for 15 min at 12,000 *g* and 4°C, and the contents of the mixing tube were transferred to a fresh RNase-free tube along with 600 *μ*L of the colorless, aqueous phase in which the RNA is located. A similar volume of 70% ethanol was added to a vortex along with the RNA mixture. Following that, the precipitate was carefully discarded and dried at room temperature, and the pellet was dissolved in 200 *μ*L of RNase-free water before being thoroughly mixed with the total RNA solution. Using the Prime Script RT kit, complementary DNA (cDNA) was created (Takara, Dalian, China). The SYBR Green real-time PCR amplification kit was used for quantitative real-time PCR according to the manufacturer's instructions. The relative expression levels of mRNA were adjusted to GAPDH. The precise primer sequences are indicated in Table 1.

### 2.10. Flow Cytometric Analysis

Liver tissue was pulverized and digested with 0.05% IV collagenases at 37°C. The supernatant was acquired by centrifuging the liver samples at 50 *g* for 3 min. The precipitate was then produced by centrifuging the supernatant at 300 *g* for 5 min and resuspending it in phosphate buffer saline. After that, liver mononuclear cells in the precipitates were treated for an hour at 4°C in the dark with antibodies labeled with PE for anti-Ly6G, APC for anti-F4/80, and FITC for CD11b. And then, add 1 *µ*L of Fixable Viability Dye eFluor™ 450(FVD) per 1 mL of cells and vortex immediately. Incubate for 30 min at 2–8°C, protect from light. Wash cells 1–2 times with phosphate buffer saline. Flow cytometry made it possible to identify the recruitment of neutrophils (Ly6G^+^/CD11b) and macrophages (F4/80^+^/CD11b^+^).

### 2.11. Western Blot

In a prechilled tube on ice, add the single-use cocktail at 10 *µ*L/mL directly to the lysis buffer. Then 100 mg of the liver was added together with 500 *μ*L of freshly prepared RIPA lysis solution containing protease inhibitors. After that, the tube was centrifuged at 4°C for 5 min at 12,000 *g* to collect the supernatant. The BCA protein detection kit was used to identify the levels of total protein. In the following step, 20 *μ*g of total protein per well was loaded into the gel. Proteins were electrophoresed through polyacrylamide-sodium dodecyl sulfate gel and transferred to a polyvinylidene fluoride membrane. The membranes were blocked with a 5% solution of BSA for 1 hr at room temperature and incubated with diluted primary antibodies overnight at 4°C. Then, the membranes were incubated with HRP-conjugated secondary antibodies. In the end, antibody binding was seen with an ECL chemiluminescent system and assessed using Image Lab software.

### 2.12. ELISA for HMGB1, TNF-*α*, IL-1*β*, and IL-10

Hepatic levels of TNF-*α*, IL-1*β*, and IL-10, and serum levels of HMGB1 were analyzed using the commercially available ELISA kits according to the manufacturer's instructions. In short, samples and standards are added to microwells coated with antimouse antibodies to incubate for 2 hr at 37°C. Then, the secondary antibody conjugated with biotin was sequentially added to the microwells to incubate for 1 hr at 37°C. After the last wash, added 100 *μ*L of HRP-avidin to each well to incubate for 1 hr at 37°C, then added 90 *μ*L of TMB substrate to each well. Finally, a colored substrate solution reactive with HRP was detected to calculate cytokines concentration.

### 2.13. Statistical Analysis

All data are expressed as the mean (Mean) ± standard deviation (SD) obtained from at least three independent repeated experiments. One-way analysis of variance was used to compare the results of different groups, and Student's *t*-test was used to compare the results of two samples. When *P* < 0.05, the difference was considered to be statistically significant; when *P* < 0.01, the difference was considered very significant. All statistical analyses were performed by using GraphPad Prism version 9 software.

## 3. Results

### 3.1. Sesamin Attenuated APAP-Induced Acute Liver Injury in Mice

The serum ALT and AST activities were examined to determine whether sesamin might attenuate liver damage in APAP-induced ALI mice. As expected in Figures 1(b) and 1(c), the ALT and AST activities were significantly higher in the APAP group than in the control group. Sesamin pretreatment, however, reduced serum ALT and AST activities in APAP-primed mice in a dose-dependent manner. Furthermore, H&E staining and quantitative analysis of hepatic injury revealed that APAP led to severe hepatic lobules necrosis, which was mitigated by sesamin pretreatment, particularly at doses of 100 mg/kg (Figures 1(d) and 1(e)).

### 3.2. Sesamin Alleviated APAP-Induced Hepatic Cellular Apoptosis in Mice

**Figure 2:**
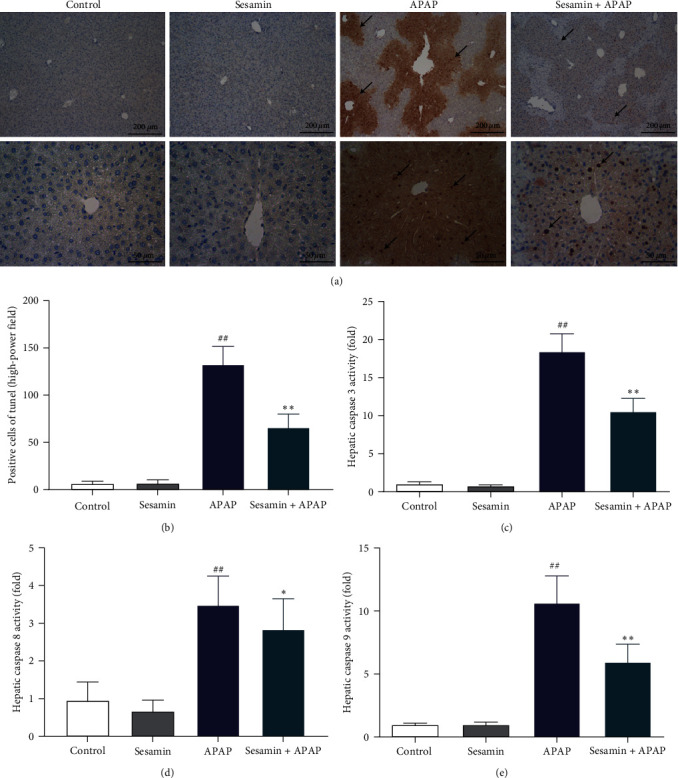
Sesamin attenuated APAP-induced hepatic cellular apoptosis in mice: (a) representative images of TUNEL staining, black arrows indicate the apoptotic hepatocytes (brown staining for the nuclei); (b) quantification of TUNEL-positive hepatocytes; (c) hepatic caspase three activity, one-fold is the amount of enzyme that will cleave 1.0 nmol of the colorimetric substrate Ac-IETD-pNA per hour at 37°C under saturated substrate concentrations; (d) hepatic caspase eight activity; (e) hepatic caspase nine activity. Data were expressed as mean ± SD; sesamin group mice were oral gavage with 100 mg/kg sesamin, APAP group mice were intraperitoneally injected with 350 mg/kg APAP for 24 hr, sesamin + APAP group mice received oral gavages of sesamin 100 mg/kg at 16.5, 8.5, and 0.5 hr for three times before 350 mg/kg APAP administration (biological repeats *n* = 6), ^##^*P* < 0.01 compared with the control group,  ^*∗*^*P* < 0.05,  ^*∗∗*^*P* < 0.01, compared with APAP group.

TUNEL and the activities of caspase-3, -8, and -9 were carried out to determine whether sesamin had an impact on APAP-induced hepatic cellular apoptosis. As shown in Figures 2(a) and 2(b), compared with the control group, APAP administration exerted a significant increase TUNEL positive cells, indicating that APAP-induced hepatic cellular apoptosis, including hepatic parenchymal and nonparenchymal cells. Moreover, pretreatment sesamin (100 mg/kg) remarkably inhibited APAP-induced apoptosis. Likewise, the activities of caspase-3, -8, and -9 in the liver were significantly increased by APAP. Pretreatment with sesamin significantly inhibited these caspases' activities in the liver of mice primed by APAP (Figure 2(c)–2(e)).

### 3.3. Effects of Sesamin on the Hepatic CYP2E1, APAP-CYS, and GSH in Mice Primied by APAP

**Figure 3:**
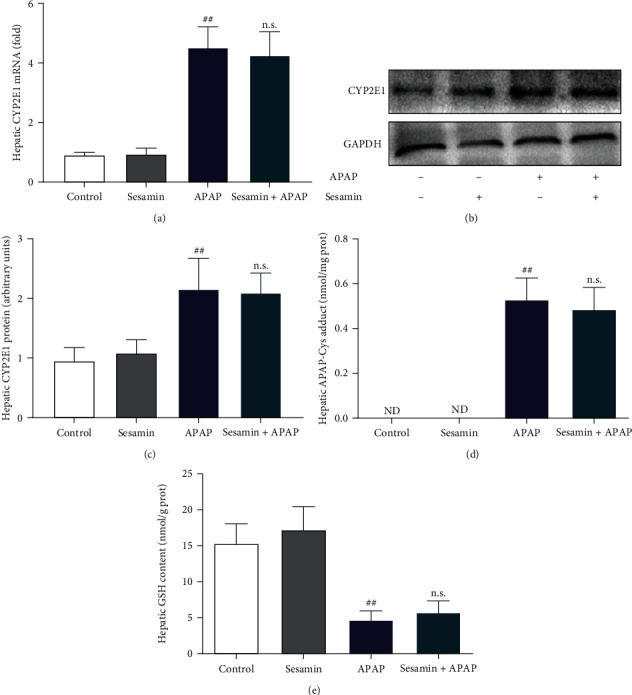
Effects of sesamin on the CYP2E1, protein adduct, and GSH caused by APAP: (a) hepatic CYP2E1 mRNA; (b) hepatic CYP2E1 protein expression; (c) quantification of hepatic CYP2E1 protein expression; (d) hepatic APAP-CYS was analyzed by HPLC-ECD; (e) hepatic GSH content. Data were expressed as mean ± SD; sesamin group mice were oral gavage with 100 mg/kg sesamin, APAP group mice were intraperitoneally injected with 350 mg/kg APAP for 24 hr, sesamin + APAP group mice received oral gavages of sesamin 100 mg/kg at 16.5, 8.5, and 0.5 hr for three times before 350 mg/kg APAP administration (biological repeats *n* = 6), ^##^*P* < 0.01 compared with the control group, no statistical difference (n.s.) compared with APAP group.

During the metabolic phase, most APAP is metabolized in the second phase by binding sulfates and glucuronides to inactivate, and a small part is oxidized by the cytochrome P450 enzyme. CYP2E1 converts APAP into NAPQI; however, the formation of NAPQI exceeds the detoxification ability of GSH, and it will covalently bind with the protein sulfide group to form the APAP-CYS protein adduct [28, 29]. Hence, we first examined the levels of CYP2E1 mRNA and protein expression. The APAP group had an increase in the expression of CYP2E1 mRNA and protein compared with the control group, but sesamin pretreatment did not affect APAP-induced expression of CYP2E1 mRNA and protein (Figure 3(a)–3(c)). Similarly, the HPLC-ECD analysis showed that there was no statistical difference in the amount of APAP-CYS between the the APAP group and the sesamin pretreatment +APAP group (Figure 3(d)). Besides, there was also no statistically significant difference in hepatic GSH content between APAP group and sesamin pretreatment +APAP groups (Figure 3(e)). The above results showed that pretreatment of sesamin has no significant effect on the expression of CYP2E1, contents of APAP-CYS, and GSH in the liver of mice administered by APAP, indicating that sesamin did not mitigate CYP-mediated APAP metabolic responses.

### 3.4. Sesamin Inhibited APAP-Induced Hepatic Oxidative Stress in Mice

**Figure 4:**
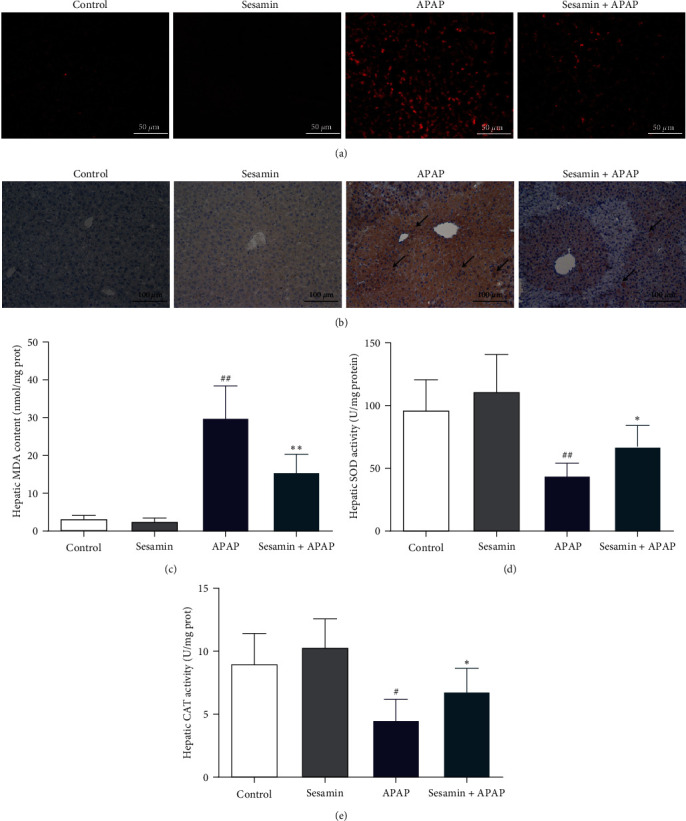
Effect of sesamin on APAP-induced oxidative stress in mice: (a) hepatic DHE fluorescence detection (Ex/Em:535/610 nm, red fluorescent marker for the nuclei indicates ROS positive); (b) the expression of hepatic 3-NT was analyzed by IHC (black arrows indicates 3-NT positive); (c) hepatic MDA content; (d) hepatic SOD activity; (e) hepatic CAT activity. Data were expressed as mean ± SD; sesamin group mice were oral gavage with 100 mg/kg sesamin, APAP group mice were intraperitoneally injected with 350 mg/kg APAP for 24 hr, sesamin + APAP group mice received oral gavages of sesamin 100 mg/kg at 16.5, 8.5, and 0.5 hr for three times before 350 mg/kg APAP administration (biological repeats *n* = 6), ^#^*P* < 0.05, ^##^*P* < 0.01 compared with the control group,  ^*∗*^*P* < 0.05,  ^*∗∗*^*P* < 0.01, compared with APAP group.

In order to elucidate hepatic oxidative stress, the levels of ROS were detected by DHE fluorescent probe. As shown in Figure 4(a), the ROS (red fluorescent marker) in the APAP group was increased compared with the control group, while the hepatic ROS was reduced in the APAP+ sesamin pretreatment group compared with the APAP group. Moreover, previous studies have shown that 3-NT, a specific marker of protein nitrous damage, increased significantly during severe oxidative stress and finally led to cell death or apoptosis [30]. Thereby, using immunohistochemical staining, we found that APAP exposure led to a massive 3-NT positive area in the liver, which was reduced by sesamin pretreatment (Figure 4(b)). Similarly, hepatic MDA content, the production of the lipid peroxidation, was obviously elevated by APAP, pretreatment with sesamin notably inhibited APAP-elicited MDA content (Figure 4(c)). In addition, the activities of antioxidative enzymes SOD and CAT in the liver were decreased by APAP administration; sesamin obviously increased the activities of the enzymes (Figures 4(d) and 4(e)).

### 3.5. Sesamin-Suppressed APAP-Induced Hepatic Proinflammatory Cytokines but Increased Anti-Inflammatory Cytokine in Mice

**Figure 5:**
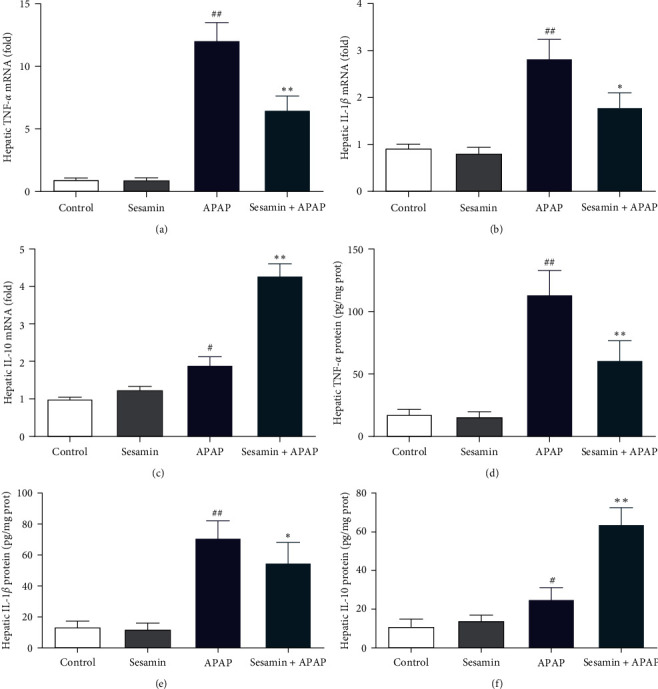
Effect of sesamin on the production of proinflammatory and anti-inflammatory cytokines in the liver of mice exposed by APAP: (a) quantification of hepatic TNF-*α* mRNA expression; (b) quantification of hepatic IL-1*β* mRNA expression; (c) quantification of hepatic IL-10 mRNA expression; (d) quantification of hepatic TNF-*α* protein levels; (e) quantification of hepatic IL-1*β* protein levels; (f) quantification of hepatic IL-10 protein levels. Data were expressed as mean ± SD; sesamin group mice were oral gavage with 100 mg/kg sesamin, APAP group mice were intraperitoneally injected with 350 mg/kg APAP for 24 hr, sesamin + APAP group mice received oral gavages of sesamin 100 mg/kg at 16.5, 8.5, and 0.5 hr for three times before 350 mg/kg APAP administration (biological repeats *n* = 6), ^#^*P* < 0.05, ^##^*P* < 0.01 compared with the control group,  ^*∗*^*P* < 0.05,  ^*∗∗*^*P* < 0.01, compared with APAP group.

To assess hepatic inflammatory response, RT-QPCR and ELISA were used to determine the levels of proinflammatory such as TNF-*α* and IL-1*β* and anti-inflammatory cytokines, such as IL-10.  Figure 5(a)–5(c) from RT-QPCR illustrated that compared with the control group, the levels of cytokines TNF-*α*, IL-1*β*, and IL-10 mRNA in the liver were significantly increased in the APAP group. However, sesamin pretreatment significantly decreased the levels of proinflammatory cytokines TNF-*α* and IL-1*β* mRNA but remarkably increased IL-10 mRNA levels in the liver of mice exposure by APAP. In line with these results obtained from RT-QPCR, the similar observations were made at the protein levels of TNF-*α*, IL-1*β*, and IL-10 in the liver (Figures 5(d) and 5(e)).

### 3.6. Sesamin Alleviated APAP-Induced Inflammatory Cells Infiltration in Mice

**Figure 6:**
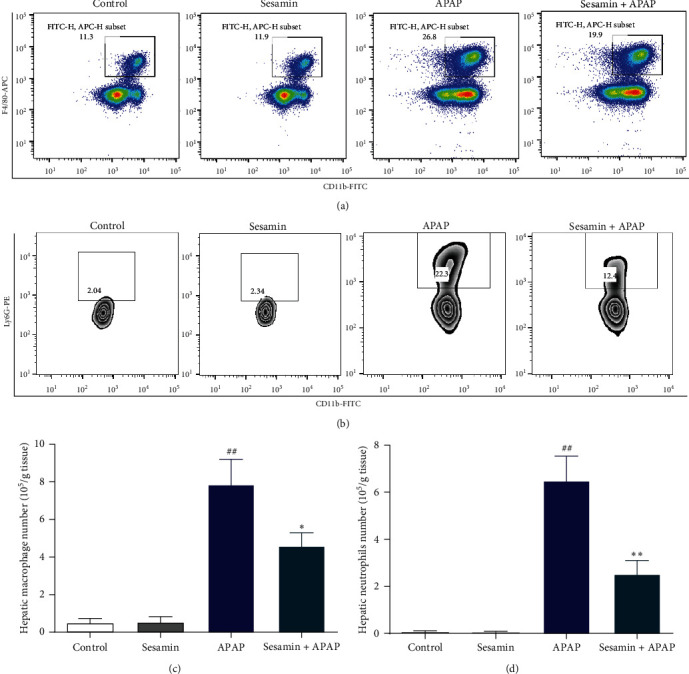
Sesamin alleviated APAP-induced hepatic inflammatory cells infiltration in mice: (a) flowchart of hepatic macrophage (CD11b^+^/F4/80^+^); (b) flowchart of hepatic neutrophil (CD11b^+^/Ly6G^+^); (c) hepatic macrophage number; (d) hepatic neutrophil number. Data were expressed as mean ± SD; sesamin group mice were oral gavage with 100 mg/kg sesamin, APAP group mice were intraperitoneally injected with 350 mg/kg APAP for 24 hr, sesamin + APAP group mice received oral gavages of sesamin 100 mg/kg at 16.5, 8.5, and 0.5 hr for three times before 350 mg/kg APAP administration (biological repeats *n* = 6), ^##^*P* < 0.01 compared with the control group,  ^*∗*^*P* < 0.05,  ^*∗∗*^*P* < 0.01, compared with APAP group.

To explore whether sesamin affects the recruitment of macrophages and neutrophils in the liver, flow cytometry detection of hepatic macrophages and neutrophils was explored. As shown in Figure 6(a)–6(c), sesamin pretreatment reduced the proportion of F4/80^+^/CD11b^+^ macrophages caused by APAP exposure, which was reduced from 26.8% to 19.9%. Meanwhile, the number of F4/80^+^/CD11b^+^ positive macrophages in the liver was significantly reduced by sesamin pretreatment when compared with the APAP group. Likewise, APAP induced an increase in the proportion and number of neutrophils (Ly6G^+^/CD11b^+^) recruitment into the liver, which was decreased by sesamin pretreatment (Figure 6(b)–6(d)).

### 3.7. Sesamin Blunted APAP-Induced HMGB1/TLR4/NF-*κ*B Activation in Mice

**Figure 7:**
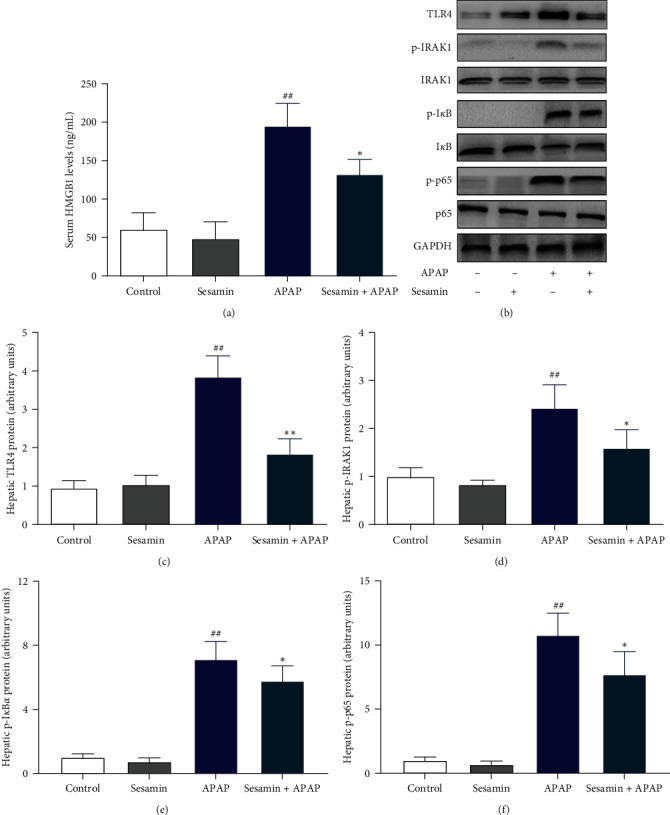
Sesamin blunted APAP-induced HMGB1/TLR4/NF-*κ*B activation in mice: (a) serum HMGB1 level was measured by ELISA; (b) the expression of hepatic TLR4/p-IRAK1/IRAK1/p-I*κ*B/I*κ*B/p-p65/p65 were detected by western blotting; (c) quantification of hepatic TLR4 protein expression; (d) quantification of hepatic p-IRAK1 protein expression; (e) quantitative analysis of hepatic p-I*κ*B*α* expression; (f) quantitative analysis of hepatic p-p65 expression. Data were expressed as mean ± SD; sesamin group mice were oral gavage with 100 mg/kg sesamin, APAP group mice were intraperitoneally injected with 350 mg/kg APAP for 24 hr, sesamin + APAP group mice received oral gavages of sesamin 100 mg/kg at 16.5, 8.5, and 0.5 hr for three times before 350 mg/kg APAP administration (biological repeats *n* = 6), ^##^*P* < 0.01 compared with the control group,  ^*∗*^*P* < 0.05,  ^*∗∗*^*P* < 0.01, compared with APAP group.

The level of HMGB1 in the serum was determined using ELISA. According to the findings, the level of HMGB1 in the APAP group was much higher than that of the control group, which was significantly reduced by sesamin (Figure 7(a)). Western blot analysis confirmed that the proteins of TLR4, p-IRAK1, and NF-*κ*B in the liver of mice exposed to APAP were significantly upregulated when compared with the control group. As expected, sesamin significantly inhibited TLR4 protein as well as the phosphorylation of IRAK1 and NF-*κ*B, including I*κ*B and p65 (Figure 7(b)–7(e)).

### 3.8. The Therapeutic Effect of Sesamin Posttreatment on APAP-Induced Liver Injury in Mice

**Figure 8:**
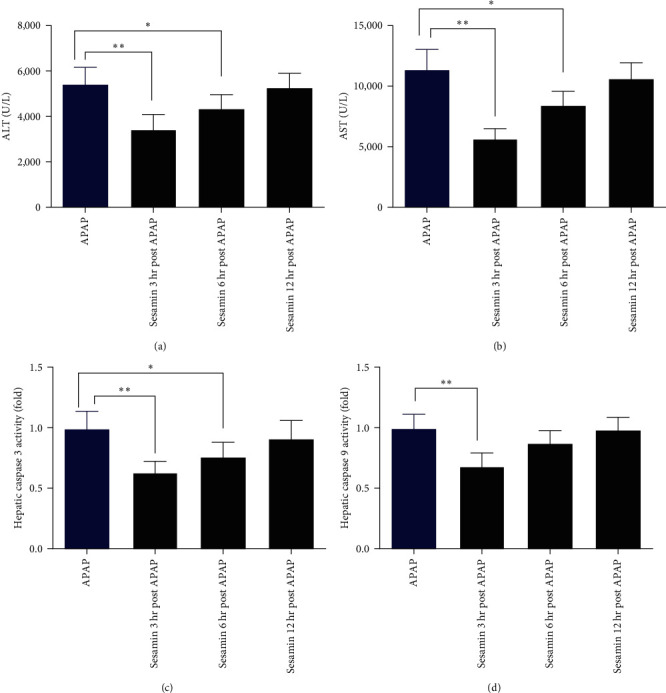
The therapeutic effect of sesamin posttreatment on APAP-induced liver injury in mice: (a) serum ALT activity; (b) serum AST activity; (c) hepatic caspase three activity; (d) hepatic caspase nine activity. Data were expressed as mean ± SD; sesamin group mice were oral gavage with 100 mg/kg sesamin, APAP group mice were intraperitoneally injected with 350 mg/kg APAP for 24 hr, sesamin + APAP group mice received oral gavages of sesamin 100 mg/kg at 16.5, 8.5, and 0.5 hr for three times before 350 mg/kg APAP administration (biological repeats *n* = 6),  ^*∗*^*P* < 0.05,  ^*∗∗*^*P* < 0.01, compared with APAP group.

To evaluate whether sesamin has a therapeutic effect after APAP, we treated mice with sesamin at 3, 6, or 12 hr after APAP exposure, respectively. The results showed that sesamin treatment at 3 or 6 hr post-APAP also could effectively attenuate the APAP-induced ALI, as indicated by reduced serum ALT and AST activities (Figures 8(a) and 8(b)) as well as hepatic caspase 3 and 8 activities (Figures 8(c) and 8(d)). However, treatment of sesamin at 12 hr after APAP administration had no obvious protective effect when compared with APAP group (Figure 8).

## 4. Discussion

APAP overdose leads to hepatotoxicity and ALF, which is urgently lack of effective agents. In this study, we found that pretreatment or posttreatment with sesamin could effectively protect mice from APAP-induced ALI and attenuated oxidative stress and inflammatory response. Notably, pretreatment of sesamin has no significant effects on the expression of CYP2E1 and contents of APAP-CYS and GSH in the liver of APAP-administered mice, indicating that sesamin did not exert a hepatoprotective effect in the APAP metabolism process. Further, we uncovered that sesamin inhibited serum HMGB1 release and hepatic TLR4 signal activation in mice exposed by APAP. The above results showed that sesamin had no significant effect on the first stage of APAP metabolism but might exert a hepatoprotective effect via inhibiting the innate immune-mediated inflammatory signal pathway.

APAP hepatotoxicity is characterized by massive oxidative stress and hepatocyte necrosis, which triggers the release of DAMPs such as HMGB1 and mtDNA, resulting in an uncontrolled inflammatory response through activating the innate immune system [31–33]. It should be noted that HMGB1 from necrotic hepatocytes plays an important role in APAP-induced ALI by activating of pattern-recognition receptors [34, 35]. Mice knockout of HMGB1 was able to reduce APAP-induced ALI compared to wild-type mice [36]. Our present results showed that APAP-triggered massive HMGB1 release, which was inhibited by sesamin pretreatment. The released HMGB1 can be recognized and bind with TLR4 on target cells, activating the NF-*κ*B signaling pathway and leading to inflammation [37]. As a result, NF-*κ*B signaling transcriptionally produced proinflammatory cytokines (TNF-*α* and IL-1*β*) and chemokines, which recruited inflammatory cells such as macrophages and neutrophils into the liver to exacerbate hepatic damage [38–40]. A previous study has found that mice-deficient TLR4 was protective from APAP toxicity [36]. Additionally, it has been demonstrated that the preservative benzyl alcohol, which is frequently added to intravenous drug solutions, can prevent ALI caused by APAP and decrease IL-1*β* release in a TLR4-dependent way. However, this medication's clinical application for treating APAP overdose in patients is constrained by its mitochondrial toxic impact [36]. In our study, ELISA and Western blot analysis confirmed that serum HMGB1 concentration and hepatic TLR4 signal were obviously elevated in APAP-exposed mice, and pretreatment with sesamin significantly reduced HMGB1 release and blocked TLR4 signal activation, resulting in limiting oxidative stress and proinflammatory cytokines (IL-1*β* and TNF-*α*) production. Interestingly, we found that sesamin exerted a remarkable increase of anti-inflammatory cytokine IL-10, which might also mediate its protection on APAP-induced ALI.

In summary, sesamin can protect mice against APAP-induced ALI by inhibiting oxidative stress and inflammatory response. The underlying mechanism might be related to inhibiting HMGB1 release, deactivation of TLR4/NF-*κ*B signaling pathway, and upregulating IL-10 production. In addition, the therapeutic time window of sesamin may reach 6 hr after APAP exposure, although its metabolism in the body remains unclear, which is related to developing a rational drug strategy on APAP hepatotoxicity.

## Figures and Tables

**Table 1:** The primers of RT-qPCR.

Target gene	Forward sequence	Reverse sequence
CYP2E1	5′-GGGGACATTCCTGTGTTCCA -3′	5′-GGATGCGGGCCTCATTA-3′
IL-1*β*	5′-ACTGTGAAATGCCACCTTTTG-3′	5′-TGTTGATGTGCTGCTGTGAG-3′
TNF-*α*	5′-CCTCCCTCTCATCAGTTCTATGG-3′	5′-CGTGGGCTACAGGCTTGTC-3′
IL-10	5′-GCTCTTACTGACTGGCATGAG-3′	5′-CGCAGCTCTAGGAGCATGTG-3′
GAPDH	5′-GTCTTCACTACCATGGAGAAGG-3′	5′-TCATGGATGACCTTGGCCAG-3′

## Data Availability

All relevant data are available from the corresponding author on request (jywan@cqmu.edu.cn).
